# Trace elements and risk of diabetes-related vascular complications: results from the EPIC-Potsdam cohort study

**DOI:** 10.1186/s12933-025-02861-y

**Published:** 2025-07-24

**Authors:** Barbaros Eroglu, Fabian Eichelmann, Olga Kuxhausf, Anna P. Kipp, Tanja Schwerdtle, Hajo Haase, Lutz Schomburg, Matthias B. Schulze

**Affiliations:** 1 Department of Molecular Epidemiology, German Institute of Human Nutrition Potsdam-Rehbruecke, 14558 Nuthetal, Germany; 2 TraceAge– DFG Research Unit on Interactions of Essential Trace Elements in Healthy and Diseased Elderly, Potsdam, Berlin, Jena, Wuppertal, Germany; 3 German Center for Diabetes Research (DZD), Neuherberg, Germany; 4 Institute of Nutritional Science, University of Potsdam, Nuthetal, Germany; 5 Department of Nutritional Physiology, Institute for Nutritional Sciences, Friedrich-Schiller University Jena, Jena, Germany; 6 Max Rubner-Institut, Federal Research Institute of Nutrition and Food, Haid-Und-Neu-Straße 9, Karlsruhe, Germany; 7 Department of Food Chemistry and Toxicology, Technische Universität Berlin, Straße Des 17. Juni 135, 10623 Berlin, Germany; 8 Institute for Experimental Endocrinology, Charité-Universitätsmedizin Berlin, Berlin, Germany

**Keywords:** Trace elements, Type 2 diabetes, Microvascular complications, Macrovascular complications

## Abstract

**Background:**

The trace elements selenium, zinc, copper, manganese, iodine, and iron are crucial for various physiological processes, including enzymatic reactions and immune responses. Dyshomeostasis of trace elements is associated with a variety of diseases including diabetes and cardiovascular diseases. It has not been clarified whether blood trace elements associate with the risk of diabetes-related vascular complications. We aimed to investigate the prospective associations between pre-diagnosis serum levels of trace elements with vascular complications in diabetes.

**Methods:**

Participants with incident diabetes and free of micro- and macrovascular disease and with pre-diagnostic serum trace element measurements from the European Prospective Investigation into Cancer and Nutrition (EPIC)-Potsdam cohort (n = 627) were followed for microvascular and macrovascular complications (n = 212 and n = 69, respectively, median follow-up: 12.8 years). We used Cox Proportional Hazard models to investigate the associations between baseline trace element levels (per SD difference) and the risk of developing diabetes-related vascular complications. To investigate the interactions and nonlinear associations between TEs and risk of diabetes-related complications, we applied Bayesian kernel machine regression (BKMR).

**Results:**

In multivariable models, higher iodine levels were associated with higher risk of developing total vascular complications (HR per SD, 95% CI: 1.16, 1.02–1.31) and microvascular complications (1.18, 1.03–1.35). In sex-stratified analyses we observed significant positive associations between zinc and total vascular complications (1.35, 1.06–1.73) and microvascular complications (1.52, 1.15–2.02) in women, while higher zinc was associated with increased risk of macrovascular complications in men (1.33, 1.00–1.77). Copper-to-Zinc ratio was inversely associated with the risk of microvascular complications in women (0.69, 0.54–0.88), but with an increased risk in men (1.54, 1.17–2.02).

**Conclusions:**

Our findings indicate that higher serum levels of iodine measured prior to the diagnosis of diabetes are associated with higher risk of subsequent microvascular complications in diabetes, while copper-to-zinc ratio is associated with microvascular complications in a sex-specific manner.

**Supplementary Information:**

The online version contains supplementary material available at 10.1186/s12933-025-02861-y.

## Introduction

The trace elements (TE) selenium (Se), zinc (Zn), copper (Cu), manganese (Mn), iodine (I) and iron (Fe) are essential micronutrients that are integral components of enzymatic reactions, signaling pathways, immune response and metabolic processes. Zn transport into β-cells is essential for insulin production, storage and secretion, therefore altered levels of Zn is hypothesized to play a role in impaired glucose-induced insulin response [1]. Cu is a catalytic cofactor of copper-zinc superoxide dismutase (CuZnSOD) enzyme and Cu deficiency compromises several components of the oxidant defence system [2]. Serum concentrations of Cu and Zn are tightly regulated by compensatory mechanisms, however, inflammatory conditions trigger specific responses that reduce serum Zn while elevating Cu concentrations [3]. Accordingly, Cu/Zn ratio was associated with an increase in different inflammatory markers in elderly populations [4, 5]. TEs can interact with each other chemically or biologically, which in turn affects the metabolism and utilization of TEs by the human organism [6]. For instance, Cu negatively affects selenoprotein expression and activity [7]. Se status affects deiodination rate and urinary iodide loss, especially in Se deficiency [8]. Fe deficiency diminishes the effectiveness of I supplementation, while concurrent Fe supplementation can enhance the overall efficacy of the supplementation [9]. Dyshomeostasis of TEs are associated with a variety of diseases including major age-related diseases such as type 2 diabetes (T2D), cardiovascular diseases (CVD) and cancer [10].

T2D is a chronic metabolic disorder characterized by hyperglycemia, which results from defects in insulin secretion, insulin action, or both. T2D is associated with significant morbidity and mortality due to its chronic complications, which are broadly classified into microvascular (retinopathy, nephropathy, and neuropathy) and macrovascular (myocardial infarction (MI) and stroke) complications [11]. Evidence suggests that TEs may influence the risk of T2D and also its complications [12–15]. Observational studies showed that low plasma levels of Zn, Se and Fe levels were associated with CVD risk in T2D patients [16, 17] whereas high Se and Cu levels were associated with higher risk of diabetic retinopathy [18, 19]. However, previous studies investigating the associations between TEs and diabetes-related vascular complications examined a single complication only and focused on each TE separately thus did not consider the interdependence of TEs, limiting the potential to capture the effect of the interplay between TEs. In addition, most of the studies were not prospectively designed which restricts their ability to draw causal conclusions from their findings. No study has yet explored the associations between diabetes-related vascular complications and plasma levels of TEs measured before diabetes diagnosis, unaffected by the disease or its treatment, thus eliminating the risk of reverse causation bias.

In this study, we aimed to investigate the prospective associations between serum levels of pre-diagnosis TEs and their functional biomarkers (Selenoprotein P (SelenoP) and Free Zinc (Free Zn)) with diabetes-related vascular complications in the EPIC-Potsdam cohort.

## Methods

### Study design and population

**Fig. 1:**
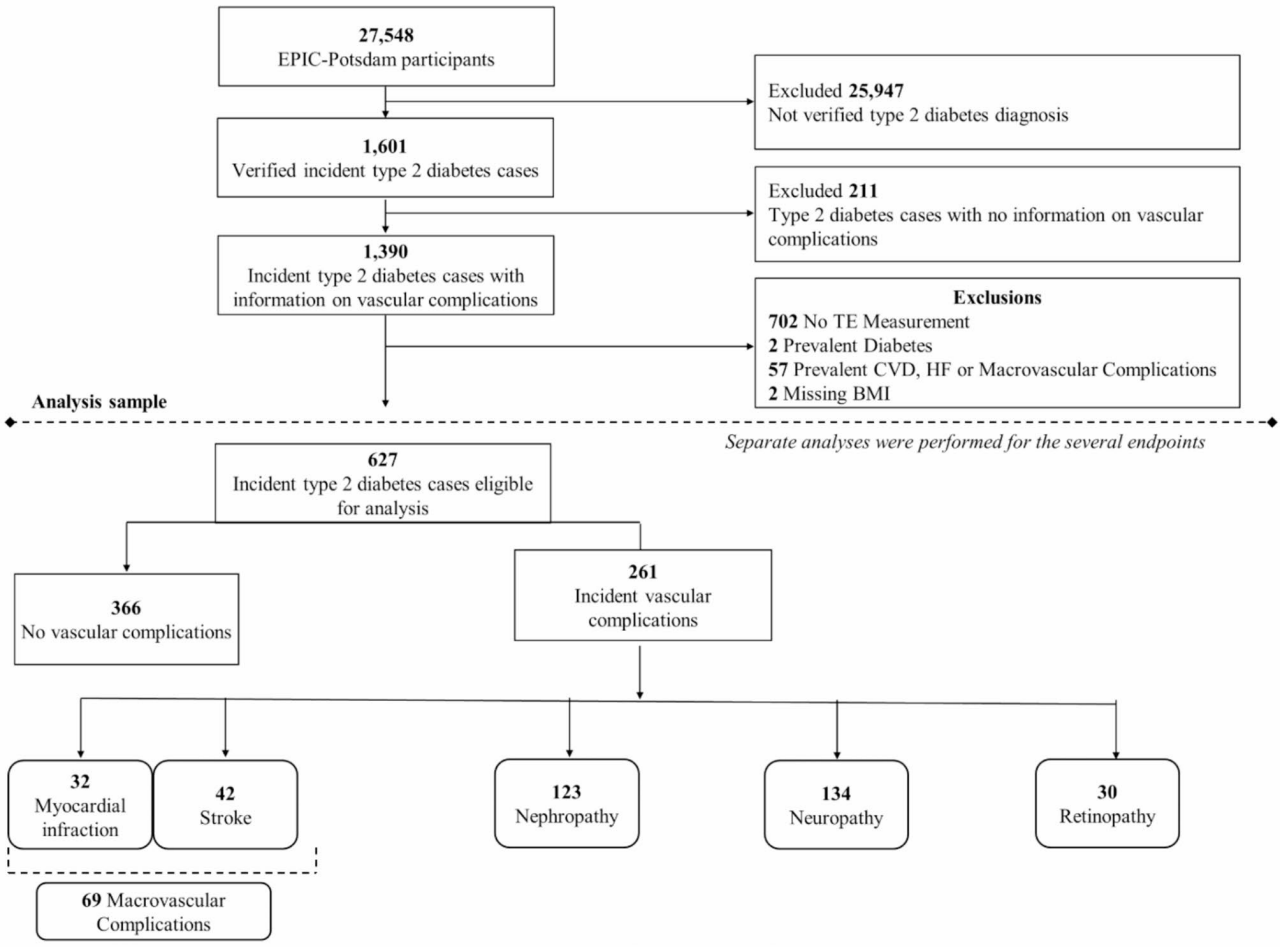
Flow chart of study sample derivation and number of microvascular and macrovascular complications of diabetes

The European Prospective Investigation into Cancer and Nutrition (EPIC)-Potsdam cohort is part of the prospective multicenter EPIC study which aims to investigate the role of nutrition in chronic diseases. 27,548 participants (16, 644 women and 10, 904 men) aged 35–64 from the general population were recruited between 1994 and 1998 in Potsdam and surrounding areas. At recruitment, participants underwent examinations that included anthropometric measurements, the collection of sociodemographic, lifestyle, dietary, and health information, as well as blood samples [20]. Follow-up on incident T2D and CVD, and lifestyle factors were conducted every 2–3 years, with response rates exceeding 90% for each follow-up round. Detailed information about recruitment and follow-up procedures has been described elsewhere [21, 22]. The analytical sample was based on all participants with incident T2D diagnosed between recruitment and January 2011 (n = 1,601), with 1,367 participants for whom information on diabetes-related vascular complications was collected. Individuals were excluded from the analyses if they had MI, stroke, heart failure, neuropathy, nephropathy or retinopathy prior to T2D diagnosis. TE measurements were performed in the context of a case-cohort design within EPIC-Potsdam, as described elsewhere [23]. This case-cohort was restricted to incident diabetes cases diagnosed until 2005, thus we excluded individuals diagnosed later without TE measurements at baseline. Participants with missing data on covariates were also excluded from the analyses. After the exclusions, the analytical sample consisted of incident T2D (n = 627) cases with available TE measurements at baseline and follow-up information on complications status from the time of T2D diagnosis through August 2017 (Fig. 1).

### Case ascertainment

Follow-up self-report questionnaires were used to identify incident diabetes cases by reported disease occurrence, diabetes-related medication use, or dietary treatment due to diabetes. Additional information was obtained from death certificates or clinical record linkage. All incident cases were verified by questionnaires sent to the physicians treating the participants. Only physician-verified T2D cases, classified based on the *International Statistical Classification of Diseases, 10th Revision* (ICD10: E11), and a diagnosis date after recruitment were included.

Information on incident diabetes-related complications was obtained through standardised forms sent to treating physicians of all incident diabetes cases. The forms collected information regarding the most recent clinic visit, and occurrence and dates of macro- and microvascular complications from the time of diabetes diagnosis until 2017. Incident macrovascular events were also ascertained from the regular follow-up of participants, following the same procedure as diabetes ascertainment. Only physician-reported or verified diabetes-related complications with a diagnosis date were included.

Microvascular complications consisted of diabetic kidney disease (ICD-10 E11.2; unspecified diabetes-related nephropathy, renal replacement therapy or albuminuria), retinopathy (ICD-10 E11.3; proliferative, non-proliferative retinopathy, or blindness) and neuropathy (ICD-10 E11.4; unspecified diabetes-related peripheral neuropathy, amputation, loss of sensation or diabetic foot syndrome). Macrovascular complications were defined as MI (ICD-10 I21) or stroke (ICD-10 I60, I61, I63, I64).

### Baseline characteristics

Sociodemographic and lifestyle characteristics were collected using self-administered and interviewer-based questionnaires at recruitment [20]. Information on smoking, leisure time physical activity, medication use and educational attainment were assessed using these questionnaires. Educational attainment was categorized as no degree/vocational training, technical college or university. Physical activity was calculated as the mean time spent on sports, biking and gardening per week.

Anthropometric measurements of participants were obtained by trained personnel at the baseline [20]. BMI was calculated as weight (kg) divided by the square of the height (m). Waist circumference was measured midway between the lower rib margin and the superior anterior iliac spine to the nearest 0.5 cm with a non-stretching tape applied horizontally.

Hypertension was defined as systolic BP ≥ 140 mmHg, diastolic BP ≥ 90 mmHg, self-reported hypertension diagnosis, or use of antihypertensive medication. Dyslipidemia was defined at recruitment as lipid-lowering medication use or prior diagnosis of dyslipidemia.

Information on the amount and frequency of food and beverage intake, and supplement use was collected at baseline via validated food frequency questionnaires described elsewhere [24]. A diet score (based on the Mediterranean diet score adapted to non-Mediterranean populations) was computed to take into consideration overall healthy diet as a determinant of TE status. Construction of this score has been documented elsewhere [25].

### Laboratory analysis

A sample of 30 mL peripheral venous blood was drawn from each participant at baseline. Blood was separated into serum, plasma (with 10% of total volume citrate) and blood cells and was subsequently stored in tanks of liquid nitrogen at -196 °C or in deep freezers at -80 °C until time of analysis. A method published previously was employed for TE profiling [26]. Briefly, 50 µL of serum sample were diluted with 440 µl of a diluent solution. As internal standard and for isotope dilution analysis 10 µL of a solution containing 50 µg/L ^77^Se and 5 µg/L Rh was added to give a total volume of 500 µL. This solution was then directly subjected to analysis via inductively coupled plasma tandem mass spectrometry (ICP-MS/MS) (Agilent ICP-QQQ-MS 8800, Agilent Technologies, Waldbronn, Germany). For external calibration of all TE except Se, standards were prepared matrix-matched in the diluent solution. Se was determined using isotope dilution analysis. For quality control, reference material RECIPE® ClinChek® serum control lyophilized (Ref. 8880–8882, Lot 347 or Lot 1497, each in both levels) was measured in triplicate daily. Mean recoveries were Mn: 98.5% ± 11.1%, Fe: 100.2% ± 7.0%, Cu: 95.5% ± 6.8%, Zn: 96.9% ± 6.1%, I: 105.9% ± 16.4%, Se: 97.3% ± 7.9%. The correlation coefficients for obtained values of serum TEs compared to reference values ranged from 0.93 to 0.99. Furthermore, sufficient blank samples (distilled H_2_O) were carried along to determine limits of detection (LOD, 3ϭ-criterion) and quantification (LOQ, 10ϭ-criterion) on a daily basis. SELENOP concentrations were quantified using a validated sandwich ELISA (selenOtest ELISA, selenOmed GmbH, Berlin, Germany), as described [27]. Free Zn concentration was determined by the low molecular weight fluorescent probe Zinypr-1 as previously reported [28], with the following modifications: The incubation times for F, F_min_ and F_max_ were set to 30, 20 and 30 min, respectively. For the induction of F_min_ 15 µL EDTA-solution (stock 800 µM, final concentration 104 µM) and for F_max_ 15 µL ZnSO_4_-solution (stock 4.5 mM; final concentration 0.52 mM) were added per well. Plasma concentrations of high-density lipoprotein cholesterol (HDL-C), total cholesterol, high-sensitivity CRP (CRP), as well as the percentage of glycated hemoglobin (HbA1c) in red blood cells were measured at the Department of Internal Medicine, University of Tübingen (Tübingen, Germany) with an automatic ADVIA 1650 analyzer (Siemens Medical Solutions, Erlangen, Germany) in 2007 [29]. Estimated glomerular filtration rate (eGFR) was calculated using the CKD-EPI formula (30). All biomarker measurements conducted in plasma were corrected for the dilution introduced by citrate volume to improve comparability with concentrations measured in EDTA-plasma reported in the literature [31].

### Statistical analysis

Demographics and laboratory characteristics were summarized as mean ± standard deviation (SD) for normally distributed variables, median with interquartile range (IQR) for non-normally distributed continuous variables and percentages for categorical variables. For Mn, 70 concentration values were below the LOD, and 59 concentration values were below the LOQ. Left-censored data were handled by substituting by LOD/√2 for censored values less than LOD and by LOQ/√2 for censored values less than LOQ. We investigated the relationships between TEs through age and sex adjusted Spearman correlations.

We performed separate analyses for total vascular complications, macrovascular complications (MI or stroke), microvascular complications (nephropathy, neuropathy or retinopathy), nephropathy and neuropathy. Due to the limited number of events, analyses for MI, stroke and retinopathy as distinct outcomes were not performed.

Cox proportional hazard models were employed to investigate the association between TE profiles and risk of macro- and microvascular complications. Participant’s age was used as underlying timescale, with entry time as age at diabetes diagnosis and exit time as age at event or censoring (date of last examination by the physician or death). The first model was adjusted for age (stratified in years) and sex. The second (main) model was further adjusted for potential confounders, including education (no degree/vocational training, trade/technical school, university degree), BMI (continuous; kg/m^2^), waist circumference (continuous; cm), smoking status (never smokers, ex-smokers, current smokers), overall leisure-time physical activity (continuous; defined as the sum of sports, biking and gardening in h/week), alcohol consumption (continuous, g/day), prevalent hypertension (yes or no), prevalent dyslipidemia (yes or no), vitamin and mineral supplement use (yes or no), dietary quality (continuous; assessed by the Mediterranean diet score) and the duration between baseline assessment and T2D diagnosis (continuous; years). We further adjusted for established vascular risk biomarkers; non-HDL cholesterol, HDL-cholesterol, hsCRP, eGFR, and HbA1c, to investigate their potential modifying effects (Model 3). In the final model (Model 4), we mutually adjusted for respective main TEs (Cu, Fe, I, Mn, Se, Zn) in addition to the covariates in the main model. Linearity of the associations were examined with restricted cubic splines, with three knots fitted at 10th, 50th, and 90th percentile of TE distribution. The associations were assessed on a continuous scale, for which a logarithmic transformation and a Z-standardization (mean = 0, SD = 1) were used to improve normality and comparability. To account for multiple comparisons, the P values were controlled for False Discovery Rate (FDR) using Benjamini–Hochberg method for each TE and outcome analysis in the main model [32]. We examined Schoenfeld residuals to validate proportional hazards assumption.

Interactions between TEs and sex were tested by creating cross product terms in the main model. We performed sensitivity analyses excluding participants who developed diabetes-related vascular complications within the first two years of diabetes diagnosis, and adjusting for menopausal status in sex-stratified analyses for total vascular complications. Further, for the associations between TEs and total vascular complications in which we observed sex differences, we created a cross product term to investigate the potential effect measure modification by menopausal status in women and ran stratified analyses by menopause status.

To investigate the joint, interactive, and nonlinear associations between TEs and risk of diabetes-related complications, we applied Bayesian kernel machine regression (BKMR) with a probit link [33]. TE concentrations were logarithmic transformed to improve normality, and we adjusted for the same covariates included in Model 2. The BKMR model was fit via a Markov chain Monte Carlo (MCMC) algorithm, running 10,000 iterations. For each TE, we computed the posterior inclusion probability (PIP) to assess its relative importance in the mixture.

A two-sided p < 0.05 denoted statistical significance in all analyses. All analyses were performed using R (version 4.3.0) statistical software.

## Results

### Participants’ characteristics

Clinical and demographic characteristics of the study participants are presented in Table 1. The median age at T2D diagnosis was 60.2 (IQR; 53.6–64.9) years and 46% of the participants were female. The median follow-up time from diabetes diagnosis on was 12.8 (IQR; 10.0–15.1) years for microvascular and 13.3 (IQR; 10.8–15.6) years for macrovascular complications. A total of 261 (42%) participants developed any vascular complications, 69 (11%) macrovascular and 212 (34%) microvascular complications. The microvascular complications were further categorized into specific types of microvascular events; 123 (20%) nephropathy events, 134 neuropathy events (21%), and 30 (5%) retinopathy events. Out of 261 participants who developed vascular complications, 104 (40%) were female.

**Table 1:** Baseline Characteristics of the Participants

Characteristic	Total, n = 627	Men, n = 341	Women, n = 286
Complication incidence	261 (42%)	157 (46%)	104 (36%)
Age at recruitment (years)^a^	56.1 (49.4, 60.6)	56.1 (49.2, 60.6)	56.4 (49.5, 60.6)
Age at T2D diagnosis (years)^a^	60.2 (53.6, 64.9)	60.0 (53.6, 64.6)	60.4 (53.7, 65.0)
Duration of diabetes^a^	12.5 (9.4, 14.9)	12.7 (9.2, 14.9)	12.3 (9.6, 14.9)
BMI (kg/m^2^)^a^	29.9 (27.4, 33.2)	29.8 (27.7, 32.3)	30.4 (27.2, 33.8)
Waist circumference (cm)^b^	100 (12.3)	104 (10.8)	94 (12.0)
Physical activity (h/week)	4.5 (1.5, 8.5)	5.0 (2.0, 9.0)	4.5 (1.0, 8.5)
Education level (%)			
Primary school	288 (46%)	139 (41%)	149 (52%)
Secondary school	155 (25%)	70 (21%)	85 (30%)
College/higher	184 (29%)	132 (39%)	52 (18%)
Smoking status (%)			
Never	233 (37%)	71 (21%)	162 (57%)
Former smoker	268 (43%)	184 (54%)	84 (29%)
Current smoker	126 (20%)	86 (25%)	40 (14%)
Alcohol intake (%)			
None	16 (2.6%)	8 (2.3%)	8 (2.8%)
Low	247 (39%)	77 (23%)	170 (59%)
Moderately low	133 (21%)	65 (19%)	68 (24%)
Moderately high	109 (17%)	84 (25%)	25 (8.7%)
High	95 (15%)	80 (23%)	15 (5.2%)
Very high	27 (4.3%)	27 (7.9%)	0 (0%)
Mediterranean diet score^b^	8.8 (2.6)	8.8 (2.6)	8.8 (2.6)
Prevalent hypertension (%)	477 (76%)	269 (79%)	208 (73%)
Prevalent dyslipidemia (%)	280 (45%)	154 (45%)	126 (44%)
Use of vitamin/mineral supplement (%)	125 (20%)	60 (18%)	65 (23%)
Copper [µg/L]^a^	1004 (868, 1183)	909 (808, 1026)	1160 (1005, 1354)
Iron [µg/L]^a^	935 (745, 1163)	962 (776, 1185)	890 (707, 1136)
Iodine [µg/L]^a^	56.7 (50.3, 66.2)	54.7 (48.1, 63.2)	59.6 (53, 68.7)
Manganese [µg/L]^a^	1.1 (0.6, 1.8)	1.2 (0.6, 1.9)	1.0 (0.5, 1.8)
Selenium [µg/L]^a^	81.0 (71.6, 92.1)	81.2 (72.0, 91.9)	80.4 (70.7, 92.4)
Zinc [µg/L]^a^	758 (672, 858)	766 (682, 869)	741 (655, 849)
Free Zinc [nM]^a^	0.6 (0.4, 0.7)	0.5 (0.4, 0.7)	0.6 (0.4, 0.8)
Selenoprotein P [mg/L]^a^	5.6 (4.7, 6.7)	5.7 (4.8, 6.8)	5.6 (4.6, 6.5)
eGFR, mL/min/1.73m^2 a^	89.8 (79.1, 98.9)	89.4 (79.0, 99.9)	91.0 (79.5, 97.8)
HbA1c, %^a^	6.1 (5.7, 6.7)	6.2 (5.8, 6.9)	6.1 (5.7, 6.6)
CRP, mg/dL^a^	0.2 (0.1, 0.4)	0.2 (0.1, 0.3)	0.3 (0.1, 0.6)
HDL-cholesterol mg/dL^a^	46.2 (39.6, 53.4)	43.3 (37.4, 49.1)	50.5 (42.4, 57.4)
Non-HDL cholesterol mg/dL^a^	165 (142, 187)	169 (145, 188)	160 (132, 183)

Data are expressed as medians (IQR)^a^ or means (SD)^b^ for continuous variables and % for categorical variables. eGFR; estimated glomerular filtration rate, HbA1c; glycated hemoglobin, CRP; C-reactive protein, HDL; high density lipoprotein

Correlations between TEs and their functional biomarkers were low to modest. The strongest correlations we observed were between Se and SelenoP (ρ = 0.45), Cu and I (ρ = 0.39), and Fe and Zn (ρ = 0.30) (Supplementary Fig. 1).

### Associations between TE levels and vascular complications

Multivariable-adjusted restricted cubic splines did not indicate non-linear associations between TEs and risk of diabetes complications (Supplementary Figs. 2–4). The Schoenfeld residuals analyses showed no evidence of a violation of the proportional hazards assumption for any exposure or outcome.

After adjustments for age, sex, education and established risk factors, higher TE concentrations at baseline were associated with higher risk of developing total vascular complications. However, none of these associations reached statistical significance, except for I (HR per SD, 95% CI: 1.15, 1.01–1.30) (Table 2, Model 2). Further adjustment for vascular risk biomarkers and other TEs did not substantially change the results (Table 2, Models 3 and 4). After performing multiple testing correction in the fully adjusted model however, the associations between I and total vascular complications did not reach statistical significance (FDR-adjusted p = 0.075) (Supplementary Table 2).

**Table 2:** Hazard ratios (95% CIs) for diabetes-related vascular complications according to TE concentrations

	Total complications		Macrovascular complications		Microvascular complications	
	N cases/ total N	HR (95% CI) per 1 SD	N cases/total N	HR (95% CI) per 1 SD	N cases/ total N	HR (95% CI) per 1 SD
Cu						
Model 1	261/627	1.05 (0.90–1.24)	69/627	1.33 (1.00–1.78)	212/624	0.98 (0.82–1.17)
Model 2	261/627	1.04 (0.88–1.23)	69/627	1.25 (0.91–1.71)	212/624	0.99 (0.81–1.21)
Model 3	240/579	0.99 (0.83–1.18)	61/579	1.22 (0.90–1.64)	198/576	0.93 (0.76–1.15)
Model 4	261/627	0.93 (0.78–1.12)	69/627	1.18 (0.83–1.67)	212/624	0.90 (0.72–1.11)
Fe						
Model 1	261/627	1.02 (0.89–1.16)	69/627	1.06 (0.81–1.38)	212/624	0.95 (0.82–1.09)
Model 2	261/627	1.04 (0.91–1.20)	69/627	1.05 (0.80–1.38)	212/624	0.97 (0.84–1.13)
Model 3	240/579	1.06 (0.91–1.23)	61/579	0.97 (0.72–1.32)	198/576	1.03 (0.87–1.22)
Model 4	261/627	1.01 (0.87–1.17)	69/627	0.95 (0.70–1.29)	212/624	0.96 (0.82–1.12)
I						
Model 1	261/627	1.15 (1.01–1.30)	69/627	1.15 (0.90–1.47)	212/624	1.14 (1.00–1.31)
Model 2	261/627	1.16 (1.02–1.31)	69/627	1.08 (0.84–1.40)	212/624	1.18 (1.03–1.35)
Model 3	240/579	1.16 (1.02–1.33)	61/579	1.02 (0.76–1.37)	198/576	1.21 (1.05–1.39)
Model 4	261/627	1.16 (1.00–1.34)	69/627	0.94 (0.70–1.25)	212/624	1.22 (1.05–1.43)
Mn						
Model 1	261/627	1.05 (0.93–1.18)	69/627	1.24 (0.98–1.56)	212/624	0.99 (0.87–1.12)
Model 2	261/627	1.02 (0.90–1.16)	69/627	1.16 (0.89–1.52)	212/624	0.97 (0.85–1.12)
Model 3	240/579	0.94 (0.83–1.08)	61/579	1.08 (0.82–1.42)	198/576	0.90 (0.78–1.05)
Model 4	261/627	1.01 (0.89–1.15)	69/627	1.17 (0.88–1.57)	212/624	0.97 (0.84–1.11)
Se						
Model 1	261/627	1.05 (0.91–1.20)	69/627	1.09 (0.85–1.39)	212/624	1.02 (0.88–1.18)
Model 2	261/627	1.07 (0.93–1.24)	69/627	1.13 (0.90–1.42)	212/624	1.04 (0.89–1.21)
Model 3	240/579	1.12 (0.97–1.29)	61/579	1.21 (0.96–1.53)	198/576	1.07 (0.92–1.26)
Model 4	261/627	1.02 (0.87–1.19)	69/627	1.06 (0.82–1.36)	212/624	0.99 (0.84–1.18)
Zn						
Model 1	261/627	1.09 (0.95–1.26)	69/627	1.27 (0.99–1.63)	212/624	1.01 (0.87–1.18)
Model 2	261/627	1.12 (0.97–1.29)	69/627	1.28 (0.99–1.66)	212/624	1.04 (0.89–1.23)
Model 3	240/579	1.12 (0.97–1.29)	61/579	1.24 (0.94–1.63)	198/576	1.09 (0.91–1.29)
Model 4	261/627	1.09 (0.93–1.27)	69/627	1.24 (0.94–1.63)	212/624	1.04 (0.86–1.24)
SelenoP						
Model 1	261/627	1.04 (0.92–1.19)	69/627	0.99 (0.78–1.26)	212/624	1.07 (0.93–1.25)
Model 2	261/627	1.03 (0.90–1.18)	69/627	1.01 (0.80–1.29)	212/624	1.06 (0.91–1.23)
Model 3	240/579	1.04 (0.90–1.21)	61/579	1.06 (0.80–1.40)	198/576	1.04 (0.89–1.22)
Free Zn						
Model 1	261/627	0.95 (0.84–1.08)	69/627	1.01 (0.81–1.25)	212/624	0.91 (0.78–1.05)
Model 2	261/627	0.94 (0.82–1.08)	69/627	1.02 (0.82–1.28)	212/624	0.89 (0.76–1.05)
Model 3	240/579	0.94 (0.81–1.08)	61/579	0.98 (0.77–1.25)	198/576	0.90 (0.76–1.06)
Se/Cu ratio						
Model 1	261/627	1.00 (0.86–1.16)	69/627	0.86 (0.66–1.13)	212/624	1.03 (0.87–1.22)
Model 2	261/627	1.03 (0.88–1.21)	69/627	0.95 (0.70–1.27)	212/624	1.04 (0.86–1.25)
Model 3	240/579	1.12 (0.94–1.34)	61/579	1.04 (0.76–1.44)	198/576	1.13 (0.93–1.39)
Cu/Zn ratio						
Model 1	261/627	0.96 (0.82–1.13)	69/627	1.01 (0.76–1.35)	212/624	0.97 (0.80–1.17)
Model 2	261/627	0.93 (0.79–1.10)	69/627	0.95 (0.71–1.26)	212/624	0.96 (0.78–1.17)
Model 3	240/579	0.89 (0.74–1.06)	61/579	0.96 (0.69–1.34)	198/576	0.87 (0.70–1.07)

TE (continuous) were log-transformed and Z-standardized (mean = 0, SD = 1)

Model 1: Adjusted for age at diabetes diagnosis, sex, Model 2 (Main Model): Model 1 + duration between recruitment and diabetes diagnosis, educational attainment, BMI, waist circumference, smoking status, physical activity, alcohol intake, vitamin and mineral supplement use, prevalent hypertension, prevalent dyslipidemia and Mediterranean diet score. Model 3: Model 2 + HDL-cholesterol, non-HDL cholesterol, HbA1c, hsCRP, eGFR. Model 4: Model 2 + respective TEs (Cu, Fe, I, Mn, Se, Zn). *SelenoP*; Selenoprotein P

With regard to macrovascular complications, higher Cu levels were associated with increased risk in the age and sex adjusted model (HR per SD, 95% CI 1.33, 1.00–1.78). However, this association was attenuated in the main model (HR per SD, 95% CI 1.25, 0.91–1.71). Higher Zn levels were also positively associated with the risk of macrovascular complications in the main model (HR per SD, 95% CI 1.28, 0.99–1.66), albeit not reaching statistical significance. We observed a positive trend in the associations between other TEs and the risk of macrovascular complications in the main models, although these associations were not statistically significant.

Higher levels of I were associated with a higher risk of developing microvascular complications (HR per SD, 95% CI 1.18, 1.03–1.35) in the main model. Positive associations were similarly observed when microvascular complications were further split into nephropathy (HR per SD, 95% CI 1.23, 1.03–1.48) and neuropathy (HR per SD, 95% CI 1.18, 1.00–1.39) (Table 3). After adjustment for multiple testing correction, the association between I and microvascular complications was not statistically significant (FDR-adjusted p = 0.092) (Supplementary Table 2).

**Table 3:** Hazard ratios (95% CIs) for diabetes-related microvascular complications by type according to TE concentrations

	Nephropathy		Neuropathy	
	N cases/ total N	HR (95% CI) per 1 SD	N cases/ total N	HR (95% CI) per 1 SD
Cu				
Model 1	123/624	1.02 (0.80–1.29)	134/624	0.96 (0.77–1.20)
Model 2	123/624	1.04 (0.79–1.36)	134/624	0.91 (0.72–1.16)
Model 3	114/576	1.01 (0.75–1.36)	124/576	0.86 (0.67–1.10)
Model 4	123/624	0.91 (0.67–1.23)	134/624	0.79 (0.61–1.04)
Fe				
Model 1	123/624	0.92 (0.77–1.10)	134/624	1.05 (0.88–1.26)
Model 2	123/624	0.96 (0.79–1.15)	134/624	1.04 (0.86–1.27)
Model 3	114/576	0.97 (0.80–1.18)	124/576	1.14 (0.91–1.42)
Model 4	123/624	0.95 (0.77–1.17)	134/624	1.03 (0.83–1.28)
I				
Model 1	123/624	1.20 (1.01–1.44)	134/624	1.20 (1.02–1.41)
Model 2	123/624	1.23 (1.03–1.48)	134/624	1.18 (1.00–1.39)
Model 3	114/576	1.24 (1.02–1.51)	124/576	1.22 (1.03–1.45)
Model 4	123/624	1.28 (1.04–1.57)	134/624	1.31 (1.07–1.60)
Mn				
Model 1	123/624	0.97 (0.82–1.15)	134/624	0.97 (0.83–1.14)
Model 2	123/624	0.94 (0.78–1.14)	134/624	0.95 (0.80–1.13)
Model 3	114/576	0.86 (0.68–1.07)	124/576	0.90 (0.75–1.08)
Model 4	123/624	0.92 (0.76–1.12)	134/624	0.92 (0.78–1.09)
Se				
Model 1	123/624	1.11 (0.93–1.32)	134/624	0.93 (0.76–1.14)
Model 2	123/624	1.10 (0.90–1.34)	134/624	0.97 (0.78–1.20)
Model 3	114/576	1.18 (0.95–1.46)	124/576	1.01 (0.81–1.25)
Model 4	123/624	1.05 (0.83–1.31)	134/624	0.91 (0.71–1.17)
Zn				
Model 1	123/624	1.03 (0.85–1.24)	134/624	1.07 (0.89–1.28)
Model 2	123/624	1.05 (0.87–1.27)	134/624	1.03 (0.85–1.26)
Model 3	114/576	1.20 (0.98–1.46)	124/576	1.04 (0.85–1.27)
Model 4	123/624	1.02 (0.81–1.28)	134/624	1.03 (0.83–1.29)
SelenoP				
Model 1	123/624	1.08 (0.88–1.31)	134/624	1.04 (0.85–1.27)
Model 2	123/624	1.05 (0.86–1.29)	134/624	1.01 (0.82–1.23)
Model 3	114/576	1.03 (0.84–1.27)	124/576	1.04 (0.84–1.29)
Free Zn				
Model 1	123/624	0.95 (0.80–1.13)	134/624	0.95 (0.77–1.18)
Model 2	123/624	0.96 (0.80–1.15)	134/624	0.95 (0.76–1.19)
Model 3	114/576	1.02 (0.85–1.22)	124/576	0.95 (0.74–1.20)
Se/Cu ratio				
Model 1	123/624	1.09 (0.87–1.38)	134/624	0.97 (0.79–1.19)
Model 2	123/624	1.07 (0.82–1.38)	134/624	1.04 (0.83–1.31)
Model 3	114/576	1.18 (0.89–1.55)	124/576	1.15 (0.90–1.47)
Cu/Zn ratio				
Model 1	123/624	0.98 (0.76–1.27)	134/624	0.91 (0.72–1.15)
Model 2	123/624	0.98 (0.74–1.29)	134/624	0.90 (0.71–1.15)
Model 3	114/576	0.81 (0.61–1.09)	124/576	0.85 (0.66–1.08)

TE (continuous) were log-transformed and Z-standardized (mean = 0, SD = 1)

Model 1: Adjusted for age at diabetes diagnosis, sex, Model 2 (Main Model): Model 1 + duration between recruitment and diabetes diagnosis, educational attainment, BMI, waist circumference, smoking status, physical activity, alcohol intake, vitamin and mineral supplement use, prevalent hypertension, prevalent dyslipidemia and Mediterranean diet score. Model 3: Model 2 + HDL-cholesterol, non-HDL cholesterol, HbA1c, hsCRP, eGFR. Model 4: Model 2 + respective TEs (Cu, Fe, I, Mn, Se, Zn). *SelenoP*; Selenoprotein P

Further adjustment for vascular risk factors and other respective TEs did not markedly change these results. Associations between other TEs and microvascular endpoints were modest and did not reach statistical significance.

The results from BKMR analyses were largely in agreement with the results from the Cox regression analyses (Supplementary Table S5, Supplementary Figs. 5–7).

**Fig. 2:**
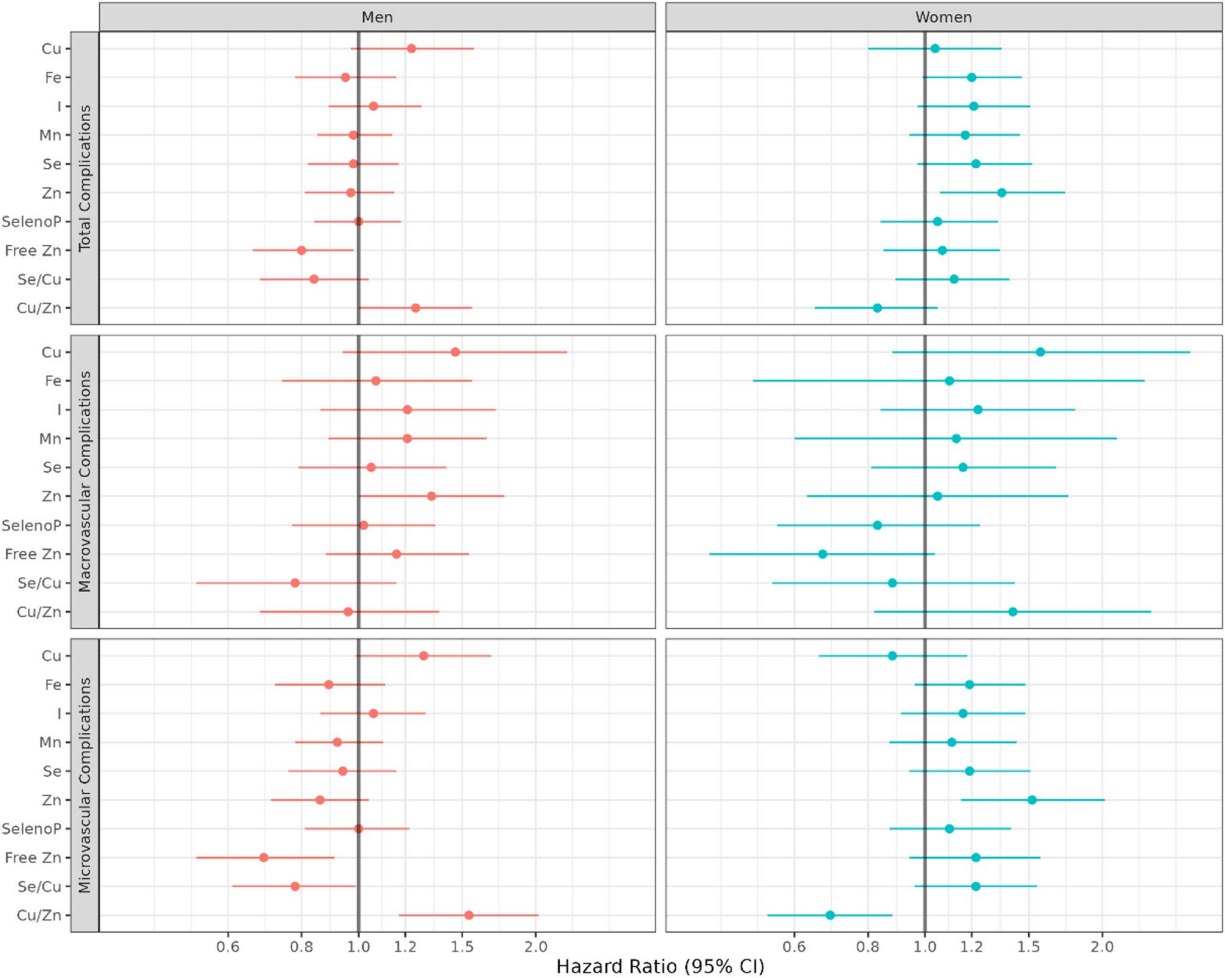
Associations between TEs and risk of diabetes related vascular complications, stratified by sex

In sex-stratified analyses (Fig. 2, Table S1), we observed a positive association between Zn and total vascular complications only among women (HR per SD, 95% CI 1.35, 1.06–1.73) while Zn levels were not associated in men (HR per SD, 95% CI 0.97, 0.81–1.15). Interaction analysis showed some evidence for heterogeneity (p interaction = 0.08). Cu/Zn ratio was positively associated with the risk of developing total vascular complications in men (HR per SD, 95% CI 1.25, 1.00–1.56), however, although not significant, the direction of the association was negative in women (HR per SD, 95% CI 0.83, 0.65–1.05). This effect modification by sex was supported by the interaction analysis (p interaction = 0.04). Investigating specific complications, we observed contrasting associations between Zn and microvascular complications risk in men and women. Higher Zn was associated with higher risk of microvascular complications in women (HR per SD, 95% CI 1.52, 1.15–2.02), however, albeit not significant, the direction of the association was the opposite for men (HR per SD, 95% CI 0.86, 0.71–1.04) (p interaction = 0.01). Cu/Zn ratio was inversely associated with the risk of microvascular complications in women (HR per SD, 95% CI 0.69, 0.54–0.88), but increased risk in men (HR per SD, 95% CI 1.54, 1.17–2.02) (p interaction = 0.001). After multiple testing correction, the observed sex-specific associations of Zn and Cu/Zn ratio with microvascular outcomes were still significant (FDR-adjusted p < 0.05). (Supplementary Table 3). We did not observe sex differences in the associations between TEs and macrovascular complications.

When we excluded participants who developed diabetes-related vascular complications within the first two years of diabetes diagnosis, higher levels of Se were associated with increased risk of macrovascular complications (HR per SD, 95% CI 1.29, 1.04–1.60). (Supplementary Table 4). Further adjustment for menopausal status in the sex-stratified analyses of total vascular complication risk did not substantially change the results (data not shown). We observed stronger associations between Zn and total vascular complications among post-menopausal women compared to pre-menopausal women, however, the interaction analysis did not indicate effect heterogeneity (p interaction = 0.40) (Supplementary Table S6).

## Discussion

In the present study, we found positive associations between serum I levels measured before the diagnosis of T2D and subsequent risk of total vascular complications, and specifically microvascular complications (both nephropathy and neuropathy). The direction of the associations between individual TEs and macrovascular complications among individuals with diabetes were positive, however, all estimates were imprecise. We observed notable sex-specific differences in certain associations between TEs and total as well as microvascular complications.

Our observation of a positive association between pre-diagnostic I levels and risk of total diabetes-related complications and, more specifically, microvascular complications seems to be in contrast to a study conducted in patients with diabetes in China, which found an inverse association between urinary I concentrations (UIC) and the risk of diabetic kidney disease [34]. However, this study was a cross-sectional study, in contrast to our prospective design. In addition, UIC is influenced by factors such as water intake and diet, and it has been suggested that, compared to UIC, serum iodine is closer to the steady state of individual iodine status [35]. Iodine is essential for the synthesis of thyroid hormones, and imbalance in iodine levels can result in thyroid dysfunction. Previous literature has shown that higher serum iodine and higher iodine intake are independently associated with higher risk of subclinical hypothyroidism (SCH) [35–37]. A meta-analysis found positive associations between SCH and the risk of developing diabetes, diabetic nephropathy and neuropathy, but not coronary heart disease [38]. Thus, the association we observed between I and microvascular complications might be mediated through SCH. We did not have the data related to thyroid hormone status, which would have allowed us to investigate this hypothesis directly.

In the age and sex adjusted model Cu was positively associated with the risk of macrovascular complications, however, adjustments for potential confounders attenuated the association. A recent meta-analysis including 17 cohort studies with more than 47,000 subjects suggested increased CVD incidence and CVD mortality risk with excessive circulating Cu levels [39]. However, two studies included in the meta-analysis, which were conducted among diabetic patients, did not find a significant association between Cu levels and incident CVD or CVD mortality [16, 40], which is in line with our finding. One possible explanation for the increased risk of CVD incidence with higher Cu levels among general population is that, Cu induces oxidative stress via its role on reactive oxygen species (ROS) production [41]. Cu is a transition metal that plays a crucial role in redox reactions, facilitating the production of ROS when present in excess exceeding the capacity of Cu chaperones [42]. Two mechanisms were proposed for Cu’s role in inducing oxidative stress. First, it can directly catalyze the formation of ROS via a Fenton-like reaction (43). Second, higher levels of Cu significantly decrease levels of glutathione, a substrate for several enzymes that remove ROS [44]. Under diabetic conditions, chronic hyperglycemia and insulin resistance promote endothelial dysfunction and low-grade inflammation, which increases the risk of CVD compared to healthy individuals [45]. This increased risk due to diabetes-related factors might explain the weaker association between Cu and CVD risk in diabetes. Furthermore, a meta-analysis of observational studies has reported that diabetes patients exhibit elevated serum Cu levels compared to healthy individuals [46]. This baseline difference could potentially mean that the impact of an increase in serum Cu on the risk of CVD incidence might be more pronounced in healthy individuals, where serum levels of Cu are relatively lower. Conversely, in diabetes patients, who already have elevated serum Cu levels, further increases may not exert as significant an influence on CVD risk as in healthy subjects.

Similar to the results from Cu analyses, we observed a non-significant positive association between pre-diagnosis Zn levels and macrovascular complications. A meta-analysis of 41 case–control studies reported that lower Zn levels were associated with higher MI risk [47]. However, in EPIC-Potsdam study, serum Zn was not associated with incident CVD risk [10]. A prospective study among 1,050 patients with diabetes observed that lower Zn levels are associated with increased risk for coronary heart disease (CHD) [48]. The design of this study differs from ours in that participants were recruited approximately eight years after their diabetes diagnosis, which may have altered their serum Zn levels. Findings from intervention trials conducted among diabetes patients suggested that beneficial effects of Zn supplementation on fasting glucose and HbA1c profiles was only observed in Zn-deficient patients defined by serum zinc concentrations below 700 µg/l [49]. In addition, a randomized controlled trial (RCT) demonstrated that Zn supplementation did not have any impact on oxidative damage and vascular function in diabetes patients with normal Zn levels [50]. Taken together, these results may indicate that the positive effect of Zn on oxidative stress and glycaemic profile in diabetes could be observed in patients with normal Zn levels, and further increases do not contribute to further improvements.

Noteworthy, we observed sex-specific associations between men and women for the association between Zn and the risk of diabetic complications. Higher Zn was associated with increased risk of macrovascular complications in men but not women, while being positively associated with total and microvascular complications in women but not men. In a case-cohort study nested within the Reasons for Geographic and Racial Differences in Stroke (REGARDS) cohort, serum Zn levels were associated with a lower risk of ischemic stroke in women but not in men [51]. A cross-sectional study reported a positive correlation between serum Zn levels and HDL-cholesterol in women but not in men [52]. Furthermore, in the Zenith study, an RCT in healthy middle-aged and older persons, Zn supplementation increased the activity of the antioxidant CuZnSOD enzyme in women, but not in men [53]. These findings might relate to the increased macrovascular complications risk in men compared to women. With regard to microvascular complications, we observed a significant positive association of Zn in women. It has been suggested that in females with diabetes, decreased levels of estradiol (E2) might increase the risk of developing renal complications [54, 55]. A cross-sectional study among 2,388 females reported a positive correlation between Cu and E2, and a weak inverse correlation between Zn and E2 [56]. Our findings of increased risk of microvascular complications in women with higher Zn, lower Cu, and lower Cu/Zn ratio, and the stronger association between Zn levels and total complications among post-menopausal women compared to pre-menopausal women might be explained by this biological mechanism. Further research into understanding sex differences in these associations is needed.

A positive association between serum Se and the risk of incident diabetes have been reported in previous meta-analyses of observational studies [12, 57], an association also being evident in the EPIC-Potsdam study [10]. An RCT reported that Se supplementation in patients with diabetes had adverse effects on blood glucose homeostasis [58]. We observed slightly increased risk of diabetic complications with higher levels of pre-diagnosis Se, though the associations were neither strong nor precise enough to draw any inference. In the sensitivity analyses where we excluded participants who developed complications within 2 years of diabetes diagnosis, we observed an increased risk of macrovascular complications with higher levels of Se. However, this result should be interpreted with caution and further studies are needed to confirm this finding given that the association was not significant after accounting for multiple testing.

It has also been suggested that lower Se levels accompanied by elevated Cu levels could increase the pro-oxidative effect of Cu [59]. Accordingly, the direction of the relationship between the Se/Cu ratio and the risk of macrovascular complications was found to be negative, which contrasts with the direction of the associations observed for Se and Cu when considered individually.

Our study has several strengths including long follow-up period, physician-verified diagnoses of T2D and of vascular complications, high response rate in follow-up for endpoints. Also, we considered multiple TEs, TE ratios and additional biomarkers, and assessed their associations with total diabetic complications and different subtypes in a relatively large sample of individuals diagnosed with T2D, although particularly the analyses on macrovascular complications lacked precision due to the relatively small number of cases. TE measurements were performed at baseline, well before the onset of diabetes. This is crucial when investigating potential causal associations between TE levels and vascular outcomes, as the concentrations are not affected by the progression of diabetes and its treatment. Additionally, we performed stratified analyses to investigate the associations within subpopulations and sensitivity analyses to ensure the robustness of our results.

Still, the study has potential limitations. Based on the observational nature, we cannot rule out the possibility of confounding. However, to address this issue, we identified potential confounders based on previous literature and adjusted for confounders in our regression models. The number of incident macrovascular complications in our sample was relatively small, which reduced the statistical power of our analyses, particularly in sex-stratified analyses. Further, there is no direct evidence of a biological mechanism that drives sex-specific associations, therefore, these associations should be interpreted with caution. TEs were measured only once, on average, 16 years prior to the development of the complications and, therefore, our measurements may not reflect long-term exposure levels well. We also do not have data on TE levels after diabetes diagnosis, thus are not able to investigate the impact of diabetes onset and different treatment regimens on TE levels. We cannot rule out the risk of inducing a collider bias by restriction on incident diabetes, however, previous studies in the same diabetes setting within the EPIC-Potsdam cohort did not indicate a strong bias [60, 61]. In addition, the parameters on the collider bias pathway must be large compared with the true causal effect for large discrepancies to occur [62].

## Conclusions

In conclusion, we identified serum I as a potential risk factor for developing diabetes-related microvascular complications, both nephropathy and neuropathy. Furthermore, our sex-stratified analyses revealed notable differences in associations, particularly for Zn and Cu/Zn ratios, demonstrating the importance of investigating sex-specific associations and considering more than one TE in disease risk analysis. Further research is required to identify potential biological mechanisms that drive these associations.

## Supplementary Information

Supplementary Information 1

## Data Availability

The datasets analyzed in the current study are not publicly available due to data protection regulations. In accordance with German Federal and State data protection regulations, epidemiological data analyses of EPIC-Potsdam may be initiated upon an informal inquiry addressed to the secretariat of the Human Study Center (Office.HSZ@dife.de). Each request will then have to pass a formal process of application and review by the respective PI and a scientific board.

## Abbreviations

BKMR: Bayesian Kernel machine regression
CHD: Coronary heart disease
CKD: Chronic kidney disease
CuZnSOD: Copper-Zinc superoxide dismutase
CVD: Cardiovascular disease
E2: Estradiol
EDTA: Ethylenediaminetetraacetic acid
EPIC: European Prospective Investigation into Cancer and Nutrition
FDR: False discovery rate
HR: Hazard ratio
ICP-MS/MS: Inductively coupled plasma tandem mass spectrometry
LOD: Limit of detection
LOQ: Limit of quantification
MI: Myocardial infarction
PIP: Posterior Inclusion Probabilities
RCT: Randomized controlled trial
ROS: Reactive oxygen species
SCH: Subclinical hypothyroidism
T2D: Type 2 diabetes
TE: Trace element
