# Supplementary material for: Trace elements and risk of diabetes-related vascular complications: results from the EPIC-Potsdam cohort study
Source: Cardiovasc Diabetol. 2025 Jul 24;24:302. doi: 10.1186/s12933-025-02861-y (PMC12291520; doi:10.1186/s12933-025-02861-y)
Supplement: Supplementary file 1 — Supplementary Information 1 [file 12933_2025_2861_MOESM1_ESM.docx]

**Trace Elements and Risk of Diabetes-Related Vascular Complications: Results from the EPIC-Potsdam Cohort Study**

**Supplementary Material**

**Supplementary Table 1** Hazard ratios (95% CIs) for diabetes-related vascular complications according to TE concentrations, stratified by sex

**Supplementary Table 2.** Unadjusted and FDR-adjusted p-values from the associations between TEs and diabetes-related vascular complications

**Supplementary Table 3.** Unadjusted and FDR-adjusted p-values from the associations between TEs and diabetes-related vascular complications, sex-stratified

**Supplementary Table 4.** Hazard ratios (95% CIs) for diabetes-related vascular complications according to TE concentrations, excluding participants developed complications within 2 years of diabetes diagnosis

**Supplementary Table 5.** Posterior Inclusion Probabilities (PIP) of TEs derived by the BKMR Model

**Supplementary Table 6.** Hazard ratios (95% CIs) and interaction analysis results for the association between Zn and total vascular complications, stratified by menopausal status

**Supplementary Figure 1.** Age and sex adjusted Spearman correlation plot of TEs, n = 627

**Supplementary Figure 2.** Restricted cubic spline regression plots for diabetes-related total vascular complications risk according to TE concentrations

**Supplementary Figure 3.** Restricted cubic spline regression plots for diabetes-related macrovascular complications risk according to TE concentrations

**Supplementary Figure 4.** Restricted cubic spline regression plots for diabetes-related microvascular complications risk according to TE concentrations

**Supplementary Figure 5.** Associations between TEs and diabetes-related total vascular complications estimated by BKMR method.

**Supplementary Figure 6.** Associations between TEs and diabetes-related macrovascular complications estimated by BKMR method.

**Supplementary Figure 7.** Associations between TEs and diabetes-related microvascular complications estimated by BKMR method.

**Supplementary Table 1.** Hazard ratios (95% CIs) for diabetes-related vascular complications according to TE concentrations, by sex

|  | **Total Complications** | | | **Macrovascular Complications** | | | **Microvascular Complications** | | |
| --- | --- | --- | --- | --- | --- | --- | --- | --- | --- |
|  | **Men** | **Women** |  | **Men** | **Women** |  | **Men** | **Women** |  |
| **N cases/ Total N** | 157/341 | 104/286 |  | 45/341 | 24/286 |  | 121/339 | 91/285 |  |
|  | **HR (95% CI) per 1 SD** | **HR (95% CI) per 1 SD** | **P-int** | **HR (95% CI) per 1 SD** | **HR (95% CI) per 1 SD** | **P-int** | **HR (95% CI) per 1 SD** | **HR (95% CI) per 1 SD** | **P-int** |
| Cu | 1.23 (0.97-1.57) | 1.04 (0.80-1.35) | 0.31 | 1.46 (0.94-2.26) | 1.57 (0.88-2.82) | 0.93 | 1.29 (0.99-1.68) | 0.88 (0.66-1.18) | 0.15 |
| Fe | 0.95 (0.78-1.16) | 1.20 (0.99-1.46) | 0.09 | 1.07 (0.74-1.56) | 1.10 (0.51-2.36) | 0.92 | 0.89 (0.72-1.11) | 1.19 (0.96-1.48) | 0.13 |
| I | 1.06 (0.89-1.28) | 1.21 (0.97-1.51) | 0.61 | 1.21 (0.86-1.71) | 1.23 (0.84-1.80) | 0.70 | 1.06 (0.86-1.30) | 1.16 (0.91-1.48) | 0.47 |
| Mn | 0.98 (0.85-1.14) | 1.17 (0.94-1.45) | 0.13 | 1.21 (0.89-1.65) | 1.13 (0.60-2.12) | 0.96 | 0.92 (0.78-1.10) | 1.11 (0.87-1.43) | 0.10 |
| Se | 0.98 (0.82-1.17) | 1.22 (0.97-1.52) | 0.11 | 1.05 (0.79-1.41) | 1.16 (0.81-1.67) | 0.48 | 0.94 (0.76-1.16) | 1.19 (0.94-1.51) | 0.12 |
| Zn | 0.97 (0.81-1.15) | 1.35 (1.06-1.73) | 0.08 | 1.33 (1.00-1.77) | 1.05 (0.63-1.75) | 0.39 | 0.86 (0.71-1.04) | 1.52 (1.15-2.02) | 0.01 |
| SelenoP | 1.00 (0.84-1.18) | 1.05 (0.84-1.33) | 0.61 | 1.02 (0.77-1.35) | 0.83 (0.56-1.24) | 0.74 | 1.00 (0.81-1.22) | 1.10 (0.87-1.40) | 0.41 |
| Free Zn | 0.80 (0.66-0.98) | 1.07 (0.85-1.34) | 0.14 | 1.16 (0.88-1.54) | 0.67 (0.43-1.04) | 0.09 | 0.69 (0.53-0.91) | 1.22 (0.94-1.57) | 0.04 |
| Se/Cu | 0.84 (0.68-1.04) | 1.12 (0.89-1.39) | 0.04 | 0.78 (0.53-1.16) | 0.88 (0.55-1.42) | 0.76 | 0.78 (0.61-0.99) | 1.22 (0.96-1.55) | 0.02 |
| Cu/Zn | 1.25 (1.00-1.56) | 0.83 (0.65-1.05) | 0.04 | 0.96 (0.68-1.37) | 1.41 (0.82-2.42) | 0.22 | 1.54 (1.17-2.02) | 0.69 (0.54-0.88) | < 0.01 |

TE (continuous) were log-transformed and Z-standardized (mean = 0, SD = 1). Adjusted for age at diabetes diagnosis, sex, duration between recruitment and diabetes diagnosis, educational attainment, BMI, waist circumference, smoking status, physical activity, alcohol intake, vitamin and mineral supplement use, prevalent hypertension, prevalent dyslipidemia and Mediterranean diet score. *SelenoP*; Selenoprotein P, *P-int*; P for interaction.

**Supplementary Table 2.** Unadjusted and FDR-adjusted p-values from the associations between TEs and diabetes-related vascular complications

|  | **Total Complications** | | **Macrovascular Complications** | | | **Microvascular Complications** | |
| --- | --- | --- | --- | --- | --- | --- | --- |
|  | **Raw p-value** | **FDR adjusted p-value** | | **Raw p-value** | **FDR adjusted p-value** | **Raw p-value** | **FDR adjusted p-value** |
| Cu | 0.658 | 0.896 | | 0.163 | 0.470 | 0.936 | 0.974 |
| Fe | 0.555 | 0.778 | | 0.715 | 0.973 | 0.735 | 0.842 |
| I | 0.021 | 0.075 | | 0.541 | 0.973 | 0.020 | 0.092 |
| Mn | 0.735 | 0.916 | | 0.274 | 0.775 | 0.714 | 0.830 |
| Se | 0.351 | 0.557 | | 0.300 | 0.818 | 0.644 | 0.773 |
| Zn | 0.127 | 0.241 | | 0.056 | 0.280 | 0.611 | 0.742 |
| SelenoP | 0.660 | 0.896 | | 0.908 | 0.973 | 0.471 | 0.679 |
| Free Zn | 0.397 | 0.576 | | 0.855 | 0.973 | 0.161 | 0.464 |
| Se/Cu | 0.693 | 0.916 | | 0.719 | 0.973 | 0.674 | 0.796 |
| Cu/Zn | 0.384 | 0.570 | | 0.715 | 0.973 | 0.656 | 0.781 |

TE (continuous) were log-transformed and Z-standardized (mean = 0, SD = 1).

Adjusted for age at diabetes diagnosis, sex, duration between recruitment and diabetes diagnosis, educational attainment, BMI, waist circumference, smoking status, physical activity, alcohol intake, vitamin and mineral supplement use, prevalent hypertension, prevalent dyslipidemia and Mediterranean diet score. *SelenoP*; Selenoprotein P

**Supplementary Table 3.** Unadjusted and FDR-adjusted p-values from the associations between TEs and diabetes-related vascular complications, sex-stratified

|  | **Total Complications** | | | | **Macrovascular Complications** | | | | **Microvascular Complications** | | | |
| --- | --- | --- | --- | --- | --- | --- | --- | --- | --- | --- | --- | --- |
|  | **Men** | | **Women** | | **Men** | | **Women** | | **Men** | | **Women** | |
|  | **Raw p-value** | **FDR Adjusted p-value** | **Raw p-value** | **FDR Adjusted p-value** | **Raw p-value** | **FDR Adjusted p-value** | **Raw p-value** | **FDR Adjusted p-value** | **Raw p-value** | **FDR Adjusted p-value** | **Raw p-value** | **FDR Adjusted p-value** |
| Cu | 0.090 | 0.256 | 0.776 | 0.911 | 0.090 | 0.300 | 0.129 | 0.291 | 0.062 | 0.280 | 0.387 | 0.630 |
| Fe | 0.601 | 0.782 | 0.069 | 0.193 | 0.723 | 0.865 | 0.804 | 0.915 | 0.300 | 0.668 | 0.112 | 0.215 |
| I | 0.510 | 0.764 | 0.086 | 0.208 | 0.265 | 0.611 | 0.281 | 0.463 | 0.590 | 0.779 | 0.227 | 0.378 |
| Mn | 0.813 | 0.894 | 0.166 | 0.347 | 0.214 | 0.545 | 0.703 | 0.825 | 0.366 | 0.668 | 0.401 | 0.631 |
| Se | 0.817 | 0.894 | 0.086 | 0.208 | 0.726 | 0.865 | 0.409 | 0.577 | 0.566 | 0.762 | 0.143 | 0.263 |
| Zn | 0.688 | 0.820 | 0.017 | 0.100 | 0.049 | 0.295 | 0.855 | 0.932 | 0.112 | 0.348 | 0.003 | 0.019 |
| SelenoP | 0.963 | 0.963 | 0.656 | 0.861 | 0.882 | 0.888 | 0.371 | 0.551 | 0.969 | 0.992 | 0.419 | 0.645 |
| Free Zn | 0.030 | 0.105 | 0.562 | 0.795 | 0.297 | 0.611 | 0.074 | 0.199 | 0.007 | 0.045 | 0.132 | 0.246 |
| Se/Cu | 0.106 | 0.275 | 0.331 | 0.533 | 0.220 | 0.550 | 0.603 | 0.728 | 0.037 | 0.216 | 0.098 | 0.199 |
| Cu/Zn | 0.053 | 0.177 | 0.120 | 0.267 | 0.832 | 0.865 | 0.211 | 0.434 | 0.002 | 0.013 | 0.003 | 0.019 |

TE (continuous) were log-transformed and Z-standardized (mean = 0, SD = 1).

Adjusted for age at diabetes diagnosis, sex, duration between recruitment and diabetes diagnosis, educational attainment, BMI, waist circumference, smoking status, physical activity, alcohol intake, vitamin and mineral supplement use, prevalent hypertension, prevalent dyslipidemia and Mediterranean diet score. *SelenoP*; Selenoprotein P

**Supplementary Table 4.** Hazard ratios (95% CIs) for diabetes-related vascular complications according to TE concentrations, excluding participants developed complications within 2 years of diabetes diagnosis

|  | | **Total Complications** | | **Macrovascular Complications** | | | **Microvascular Complications** | |
| --- | --- | --- | --- | --- | --- | --- | --- | --- |
| **N cases/ Total N** | **246/608** | | | | **59/608** | | **205/606** | |
|  | | **HR (95% CI) per 1 SD** | **FDR adjusted P-value** | | **HR (95% CI) per 1 SD** | **FDR adjusted P-value** | **HR (95% CI) per 1 SD** | **FDR adjusted P-value** |
| Cu | | 1.04 (0.88-1.23) | 0.922 | | 1.23 (0.92-1.64) | 0.468 | 1.01 (0.82-1.24) | 0.938 |
| Fe | | 1.06 (0.92-1.23) | 0.543 | | 1.04 (0.78-1.39) | 0.841 | 1.01 (0.87-1.17) | 0.931 |
| I | | 1.14 (1.00-1.30) | 0.131 | | 1.04 (0.79-1.37) | 0.841 | 1.17 (1.02-1.36) | 0.110 |
| Mn | | 0.99 (0.87-1.12) | 0.960 | | 1.03 (0.78-1.34) | 0.873 | 0.98 (0.85-1.13) | 0.902 |
| Se | | 1.11 (0.96-1.29) | 0.319 | | 1.29 (1.04-1.60) | 0.150 | 1.04 (0.89-1.23) | 0.843 |
| Zn | | 1.13 (0.98-1.30) | 0.242 | | 1.30 (0.97-1.73) | 0.380 | 1.09 (0.92-1.29) | 0.494 |
| SelenoP | | 1.08 (0.95-1.24) | 0.371 | | 1.12 (0.88-1.42) | 0.841 | 1.10 (0.94-1.27) | 0.393 |
| Free Zn | | 0.97 (0.84-1.11) | 0.888 | | 1.04 (0.81-1.34) | 0.841 | 0.92 (0.79-1.08) | 0.528 |
| Se/Cu | | 1.07 (0.91-1.25) | 0.571 | | 1.09 (0.81-1.45) | 0.841 | 1.03 (0.86-1.25) | 0.873 |
| Cu/Zn | | 0.92 (0.78-1.09) | 0.494 | | 0.96 (0.70-1.33) | 0.873 | 0.93 (0.76-1.14) | 0.710 |

^ᵻ^TE (continuous) were log-transformed and Z-standardized (mean = 0, SD = 1).

Adjusted for age at diabetes diagnosis, sex, duration between recruitment and diabetes diagnosis, educational attainment, BMI, waist circumference, smoking status, physical activity, alcohol intake, vitamin and mineral supplement use, prevalent hypertension, prevalent dyslipidemia and Mediterranean diet score. *SelenoP*; Selenoprotein P

**Supplementary Table 5.** Posterior Inclusion Probabilities (PIP) of TEs derived by the BKMR Model

|  | **Total Complications** | **Macrovascular Complications** | **Microvascular Complications** |
| --- | --- | --- | --- |
|  | **PIP** | **PIP** | **PIP** |
| Cu | 0.375 | 0.390 | 0.324 |
| Fe | 0.293 | 0.293 | 0.266 |
| I | 0.620 | 0.373 | 0.646 |
| Mn | 0.173 | 0.231 | 0.175 |
| Se | 0.348 | 0.352 | 0.245 |
| Zn | 0.333 | 0.524 | 0.303 |

**Supplementary Table 6.** Hazard ratios (95% CIs) and interaction analysis results for the association between Zn and total vascular complications, stratified by menopausal status

|  | **Pre-Menopause** | | | **Post-Menopause** | | |  |
| --- | --- | --- | --- | --- | --- | --- | --- |
|  | **N cases/Total** | **HR (95% CI) per 1 SD** | **P-value** | **N cases/Total** | **HR (95% CI) per 1 SD** | **P-value** | **P-interaction** |
| Zn | 48/125 | 1.52 (1.03-2.25) | 0.04 | 56/161 | 1.15 (0.86-1.53) | 0.36 | 0.40 |

Zn was log-transformed and Z-standardized (mean = 0, SD = 1).

Adjusted for age at diabetes diagnosis, sex, duration between recruitment and diabetes diagnosis, educational attainment, BMI, waist circumference, smoking status, physical activity, alcohol intake, vitamin and mineral supplement use, prevalent hypertension, prevalent dyslipidemia and Mediterranean diet score. *P-interaction:* P-value of the interaction test between Zn and Menopausal status.

**Supplementary Figure 1** Age and sex adjusted Spearman correlation plot of TEs, n = 627**
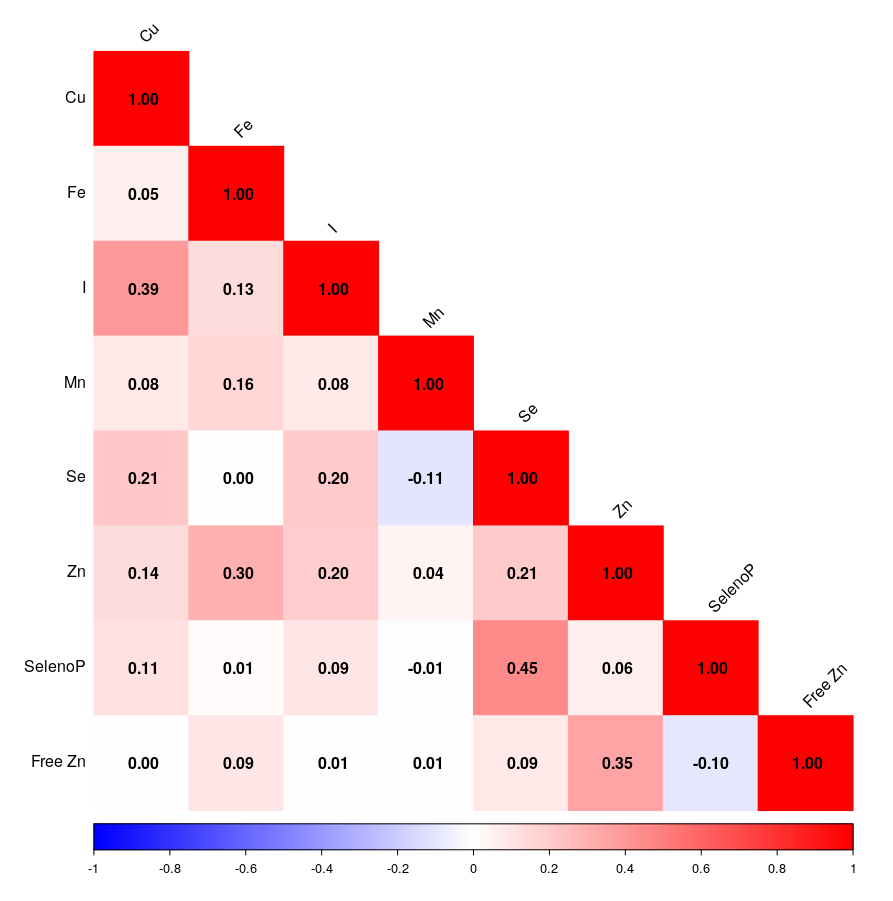
**

**Supplementary Figure 2** Multivariable adjusted hazard ratios for diabetes-related total vascular complications risk according to TE concentrations**
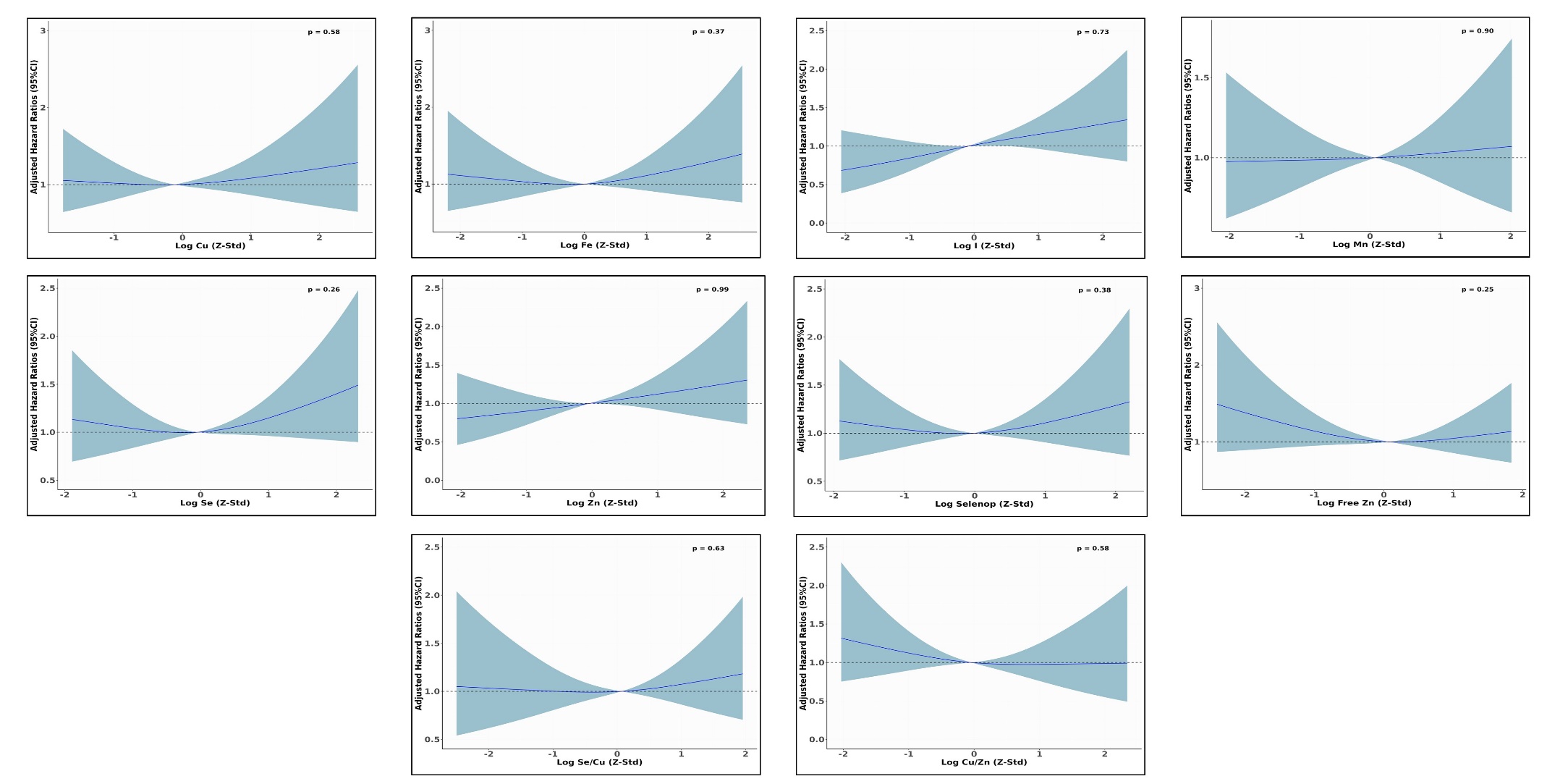
**Multivariable adjusted hazard ratios for diabetes related total vascular complications risk according to TE concentrations**.** Models were adjusted for age at diabetes diagnosis, sex, educational attainment, BMI, waist circumference, smoking status, physical activity, alcohol intake, vitamin and mineral supplement use, prevalent hypertension, anti-hypertensive medication, lipid-lowering medication and Mediterranean Diet Score. TEs were log transformed and Z standardized (Mean = 0, SD =1). Knot placement was 10^th^, 50^th^, and 90^th^ percentile. P values represent the test for nonlinearity results.

**Supplementary Figure 3** Multivariable adjusted hazard ratios for diabetes-related macrovascular complications risk according to TE concentrations
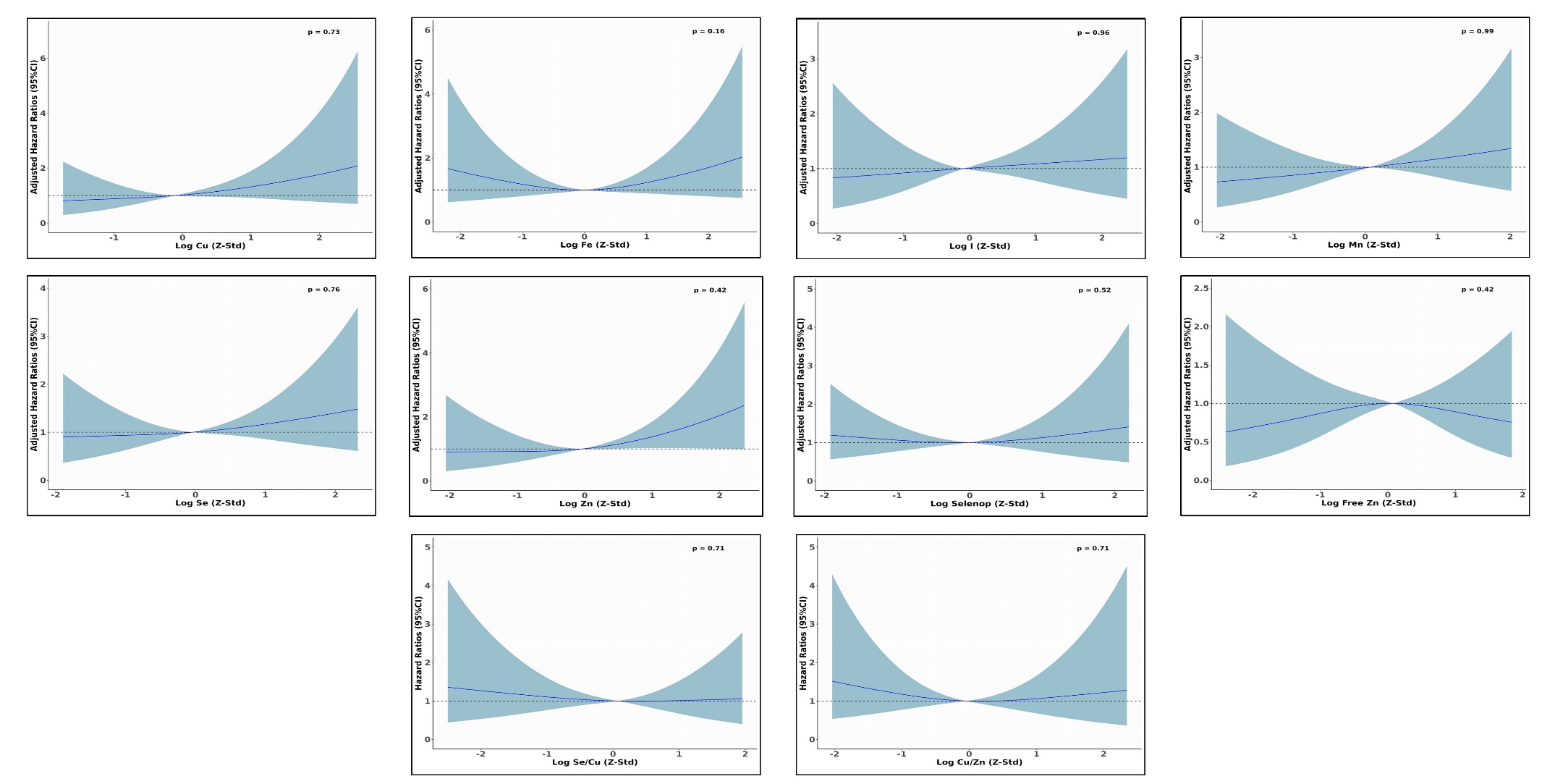


Multivariable adjusted hazard ratios for diabetes related macrovascular complications risk according to TE concentrations**.** Models were adjusted for age at diabetes diagnosis, sex, educational attainment, BMI, waist circumference, smoking status, physical activity, alcohol intake, vitamin and mineral supplement use, prevalent hypertension, anti-hypertensive medication, lipid-lowering medication and Mediterranean Diet Score. TEs were log transformed and Z standardized (Mean = 0, SD =1). Knot placement was 10^th^, 50^th^, and 90^th^ percentile. P values represent the test for nonlinearity results.

**Supplementary Figure 4** Multivariable adjusted hazard ratios for diabetes-related microvascular complications risk according to TE concentrations
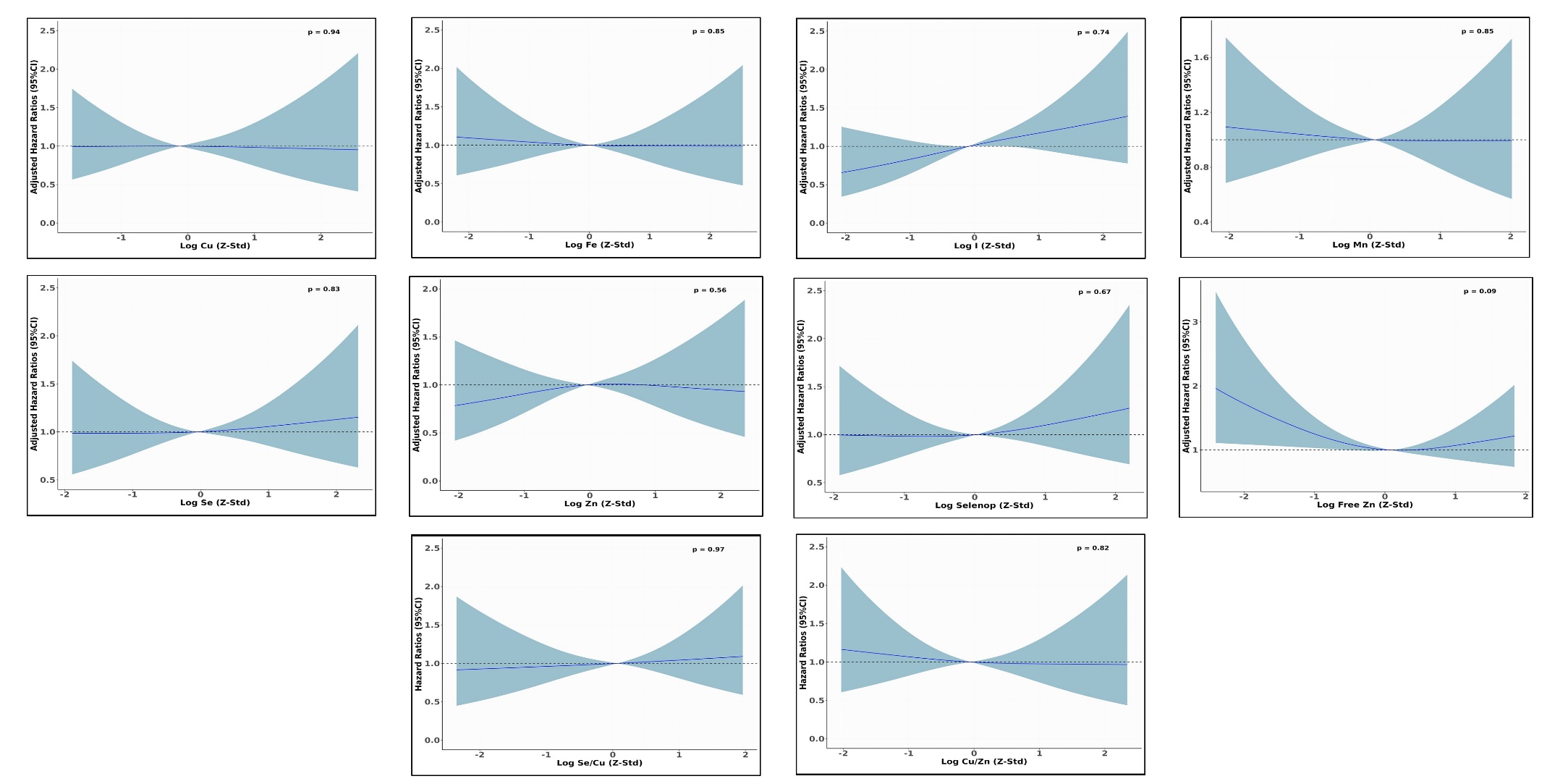


Multivariable adjusted hazard ratios for diabetes related microvascular complications risk according to TE concentrations**.** Models were adjusted for age at diabetes diagnosis, sex, educational attainment, BMI, waist circumference, smoking status, physical activity, alcohol intake, vitamin and mineral supplement use, prevalent hypertension, anti-hypertensive medication, lipid-lowering medication and Mediterranean Diet Score. TEs were log transformed and Z standardized (Mean = 0, SD =1). Knot placement was 10^th^, 50^th^, and 90^th^ percentile. P values represent the test for nonlinearity results.

**Supplementary Figure 5**. Associations between TEs and diabetes-related total vascular complications estimated by BKMR method.


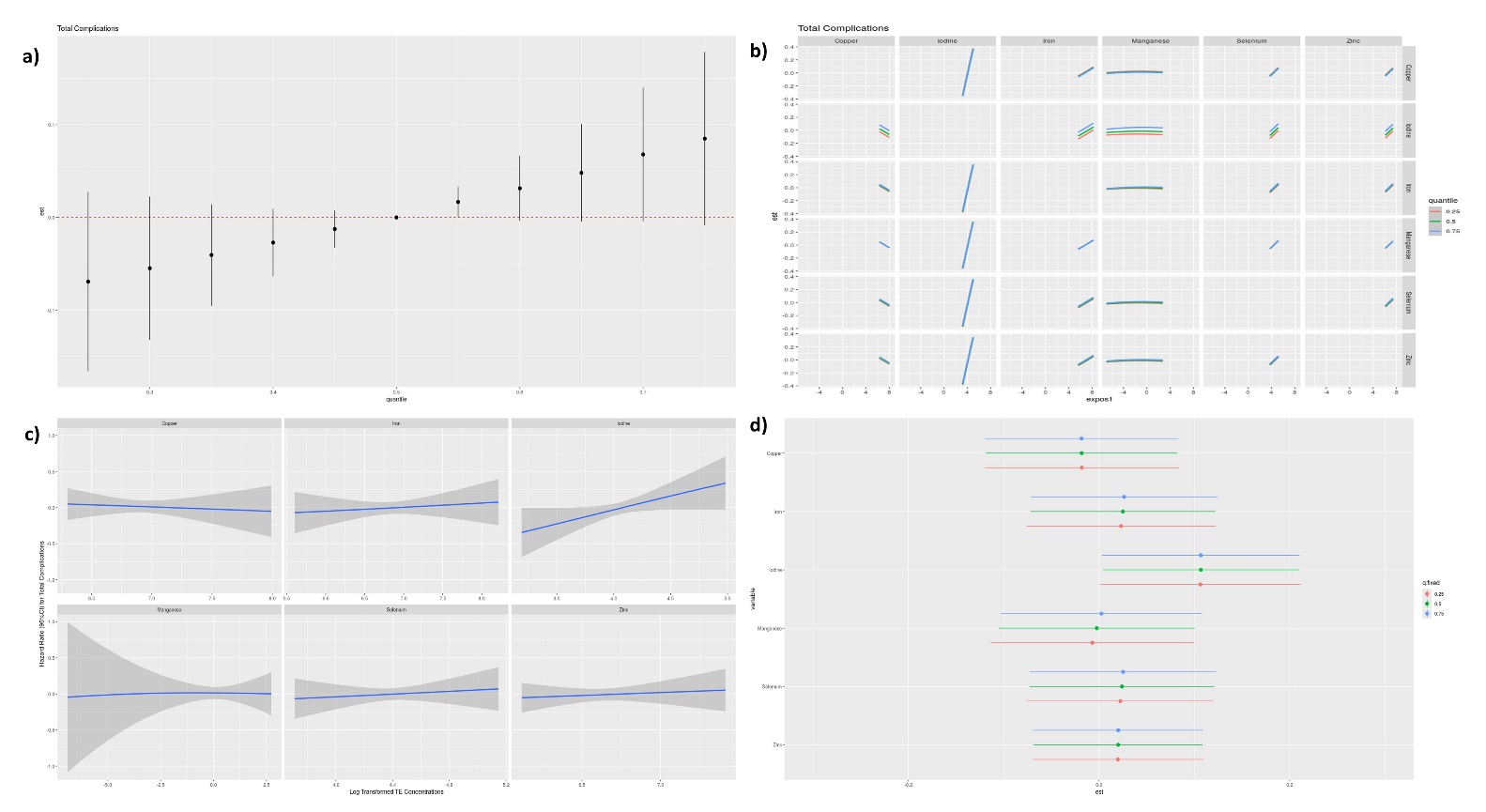


Models were adjusted for age at diabetes diagnosis, sex, duration between recruitment and diabetes diagnosis, educational attainment, BMI, waist circumference, smoking status, physical activity, alcohol intake, vitamin and mineral supplement use, prevalent hypertension, prevalent dyslipidemia and Mediterranean diet score. a) Joint effects of TE mixture with diabetes-related total vascular complications risk at increasing percentiles compared to medians b) Bivariate exposure-response functions for each TE among six TE at varying levels (10th, 50th, 90th) of another TE, when other TEs are set at their median c) Univariate exposure-response function between individual TE with the risk of diabetes-related total vascular complications with other TEs fixed at the corresponding 50th percentiles d) Single-exposure effect of individual TE for an IQR increase on the risk of diabetes-related total vascular complications when other metals are fixed at their 25th, 50th, or 75th percentiles

**Supplementary Figure 6.** Associations between TEs and diabetes-related macrovascular complications estimated by BKMR method.


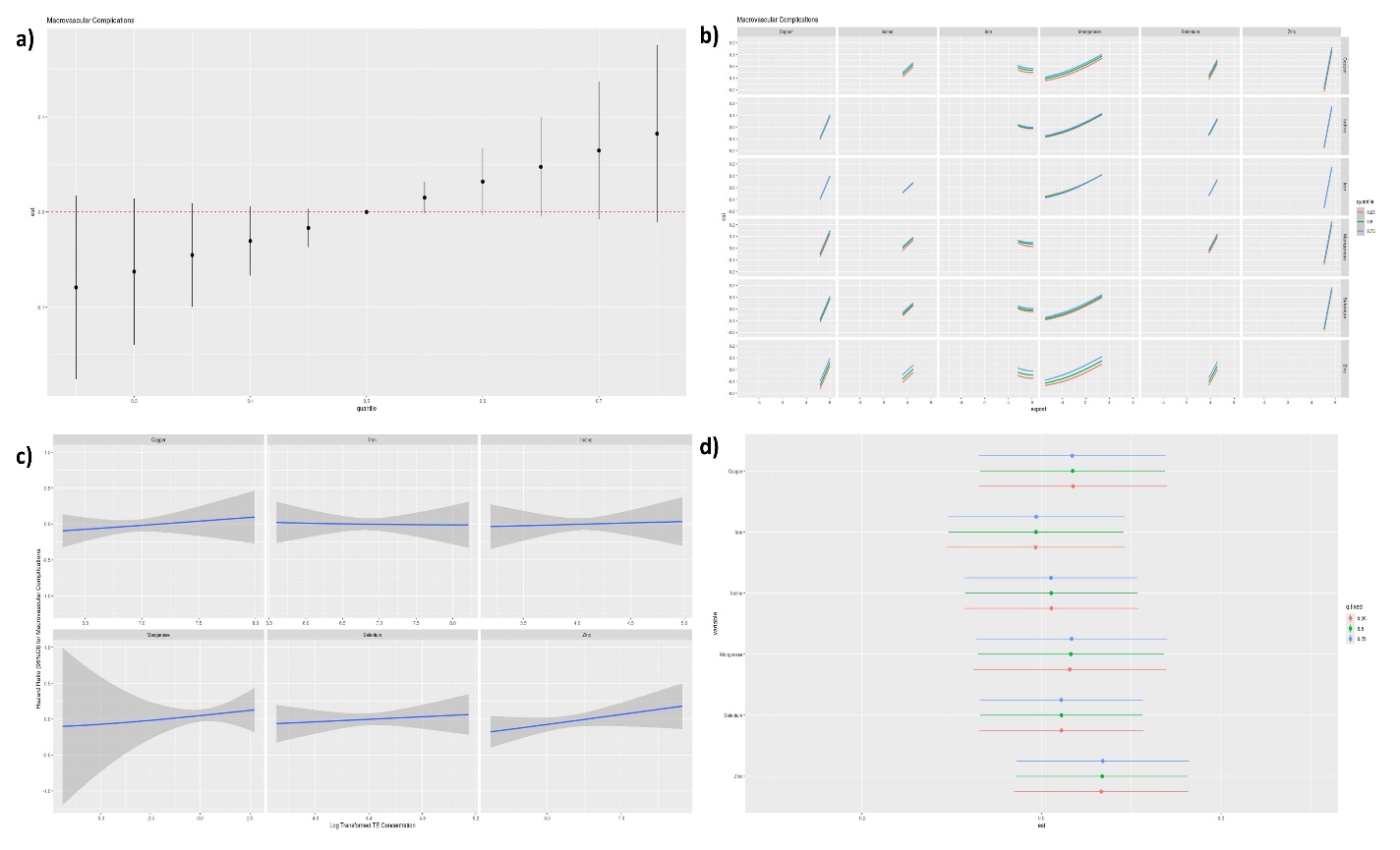


Models were adjusted for age at diabetes diagnosis, sex, duration between recruitment and diabetes diagnosis, educational attainment, BMI, waist circumference, smoking status, physical activity, alcohol intake, vitamin and mineral supplement use, prevalent hypertension, prevalent dyslipidemia and Mediterranean diet score. a) Joint effects of TE mixture with diabetes-related macrovascular complications risk at increasing percentiles compared to medians b) Bivariate exposure-response functions for each TE among six TE at varying levels (10th, 50th, 90th) of another TE, when other TEs are set at their median c) Univariate exposure-response function between individual TE with the risk of diabetes-related macrovascular complications with other TEs fixed at the corresponding 50th percentiles d) Single-exposure effect of individual TE for an IQR increase on the risk of diabetes-related macrovascular complications when other metals are fixed at their 25th, 50th, or 75th percentiles

**Supplementary Figure 7.** Associations between TEs and diabetes-related microvascular complications estimated by BKMR method.


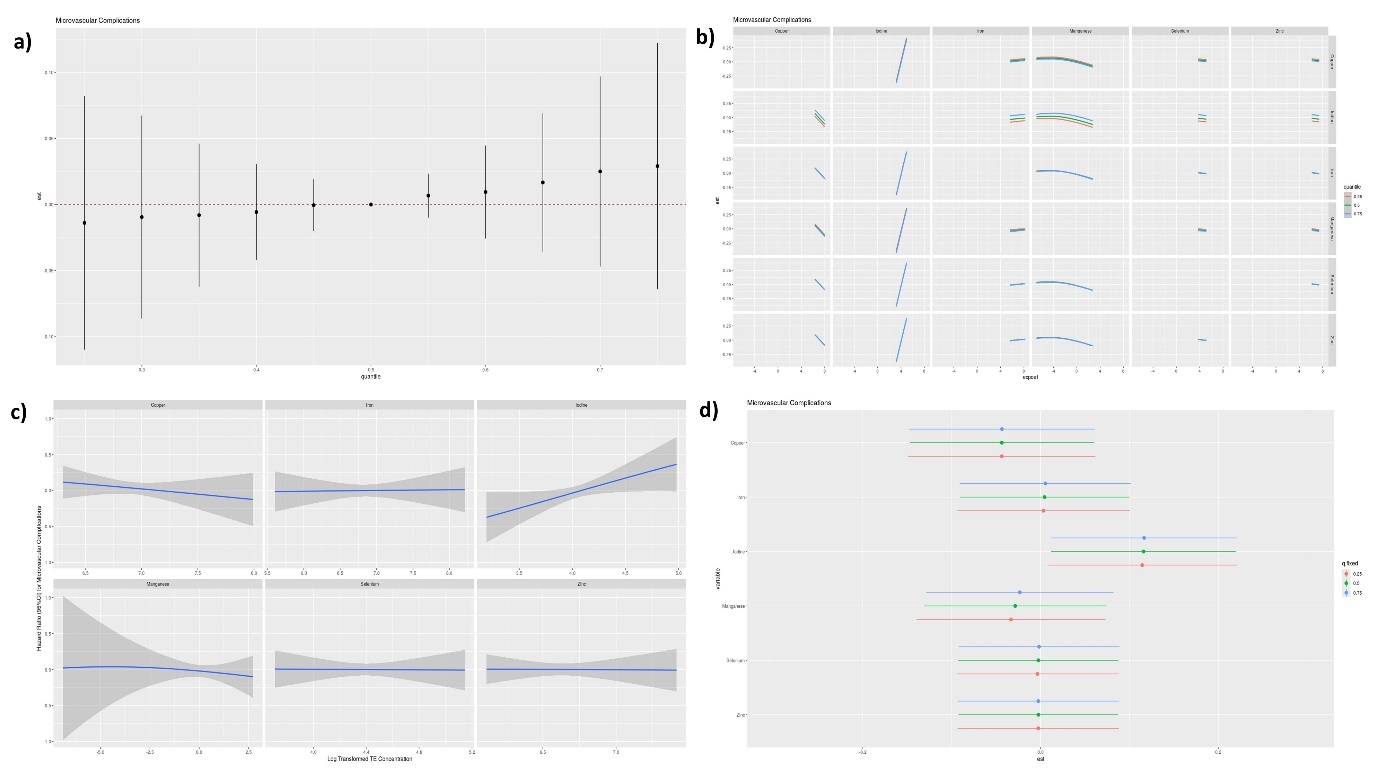


Models were adjusted for age at diabetes diagnosis, sex, duration between recruitment and diabetes diagnosis, educational attainment, BMI, waist circumference, smoking status, physical activity, alcohol intake, vitamin and mineral supplement use, prevalent hypertension, prevalent dyslipidemia and Mediterranean diet score. a) Joint effects of TE mixture with diabetes-related microvascular complications risk at increasing percentiles compared to medians b) Bivariate exposure-response functions for each TE among six TE at varying levels (10th, 50th, 90th) of another TE, when other TEs are set at their median c) Univariate exposure-response function between individual TE with the risk of diabetes-related microvascular complications with other TEs fixed at the corresponding 50th percentiles d) Single-exposure effect of individual TE for an IQR increase on the risk of diabetes-related microvascular complications when other metals are fixed at their 25th, 50th, or 75th percentiles
